# Experiences of Male Student Nurse Midwives in Malawi During Undergraduate Education

**DOI:** 10.29024/aogh.18

**Published:** 2018-04-30

**Authors:** Miriam M. Chinkhata, Gayle Langley

**Affiliations:** 1 Malawi College of Health Sciences, Private Bag 396 Chichiri Blantyre 3, MW; 2 Department of Nursing Education, Witwatersrand University, ZA

## Abstract

**Background:**

Historically, nursing has evolved from being a feminine profession to a profession accepted by both sexes. In the contemporary world, gender mainstreaming has been instituted as a global strategy in promoting gender equality. Though men continue to join nursing, they face many challenges. It is believed that through proper socialization some of the challenges can be addressed. In Malawi, there is dearth of literature on this subject. This resulted in undertaking the study.

**Objective:**

The goal of the study was to describe experiences of male student nurses during undergraduate education in Malawi.

**Methods:**

A qualitative descriptive design was utilised. Focus group discussions were conducted with study participants in purposively selected nursing colleges.

**Findings:**

Male student nurse midwives faced more negative than positive experiences in nursing. The following three major themes were generated: nursing is a feminine occupation, facing discrimination and socialisation experienced.

**Conclusion:**

Malawian male student nurse midwives face both positive and negative experiences during their integration in nursing. Formulation and implementation of gender sensitive policies would help in strengthening male nurse education.

## Introduction

Historically nursing has evolved as a feminine profession despite some men having performed caring roles since the profession’s infancy [1,2,3]. With the reforms brought about by the founder of modern nursing, Florence Nightingale, men were secluded from being trained as nurses [1,2]. Nowadays, though there is an increase in the number of men entering the nursing profession, they remain a minority in the female dominated profession [4]. This paper describes the findings of a study focusing on the experiences of male student nurses during their education in nursing in Malawi.

Modern nursing started in the United Kingdom with the founder Florence Nightingale introducing many reforms. These reforms contributed to the stereotype that resulted in the nursing profession being perceived as feminine. Nightingale’s reforms resulted in the education and training of male nurses in England not being recognised; and in most hospitals only ‘lady nurses’ were recruited [1]. Following the Second World War, a chronic shortage of nurses ensued, secondary to the expansion of alternative work opportunities for women and the growth of the general hospital sector. This led to the formal acceptance of the education and training and registration of male nurses [1].

To ensure that the traditions and dignity of the nursing profession among male nurses were upheld, The Society of Registered Male Nurses was established. However, despite these developments, male nurses faced gender-based discrimination from female nurses who believed nursing was a woman’s occupation and that the introduction of male nurses violated the nursing profession’s respect. Consequently, this affected the recruitment and retention of male nurses in England [1].

Similarly, in other countries, the trend of enrolling men into the nursing profession took place [2]. In America, South Africa and Israel, the incorporation of men into the nursing profession started due to chronic shortages of female nurses after the Second World War [2,3]. The men served mainly in the military and mine hospitals and primarily cared for the mentally ill and patients who required restraints [2,5,6]. In these countries too, segregation of men in the profession took place [2].

Given the history of men in the nursing field, various studies have been conducted to establish their experiences [7,8,9,10]. Most of these studies reported that men in nursing experience various challenges such as isolation, role strain and lack of male role models and that there is a need to provide support and counselling during the course of education and beyond.

In an effort to eliminate gender discrimination in the nursing profession, in our contemporary world, gender mainstreaming has been instituted as a major global strategy in promoting gender equality [11]. Gender equality, if properly implemented, results in effectiveness and efficiency in service delivery [12].

To promote integration of men in nursing, nursing professionals have a duty to cultivate gender competence, understand gender-related policies and guidelines as well as gender issues in education, research and decision-making processes [11].

Malawi is one of the countries that educate and train male nurse midwives. The recruitment of men started in 1985 [13] and currently two cadres of nurses are educated; Registered Nurse Midwives (RNs), and Nurse Midwife Technicians NMTs. The RNs are educated for four years and graduate with a bachelor’s degree in universities [14] while at a government nursing college they are awarded a diploma. On the other hand, NMTs, are educated for three years mainly in faith-based institutions under a Christian umbrella body called Christian Health Institution of Malawi (CHAM). The universities are located in cities unlike the CHAM nursing colleges, most of which are in the rural areas. The NMTs replaced enrolled nurses and work under the supervision of the registered nurses. All the nursing colleges in Malawi educate both male and female nurse midwives though the word ‘nurse’ is mostly utilized to mean both nurse and midwife.

According to the policy on gender in Malawi [15] an equal number of males and females should enter tertiary education. However, this is not the case with nursing. The historical background of nursing being ‘feminine,’ and the challenges men face in nursing, impact negatively on those intending to enter the profession [1,16,17,18]. In addition, anecdotal reports reveal that some men in nursing display unprofessional tendencies which could negatively influence the number of men entering and being retained in the profession in the country.

Little is known about the experiences of men in nursing during their education in Malawi as literature focusing on this phenomenon is scarce. It might be argued that international literature can inform Malawi on the experiences and best lessons could be learnt. However, some of the lessons may not be applicable to the Malawian context as education is influenced by many factors. As such, it is important to study the experiences of male student-nurse midwives during their education in the female dominated profession in Malawi; and develop tailor-made strategies to address problems that male nurse midwives may be encountering and strengthen the gains made in recruiting men into the profession. The findings should help improve the integration of male nurse midwives in the nursing profession in the country.

The goal of the study was to describe the experiences of male student nurse midwives during their education and training in nursing in Malawi.

## Methods

The study utilized a descriptive qualitative design aimed at understanding and describing the experiences of Malawian male student nurse midwives during their socialisation process in nursing in Malawi. The social role theory as expounded by Eagly and Wood [19] was utilized.

The setting for the study was the nursing education institutions in Malawi. At the time of data collection, Malawi hosted 13 nursing education institutions of which, three were universities, one government institution and the rest faith-based institutions. The study was undertaken in six purposively selected nursing colleges to ensure an appropriate representation of the various types of the colleges.

The total population of male student nurses in the six nursing colleges was 572 students (N = 572). Seventy (n = 70) male student nurse midwives undertaking a course in nursing participated in the study. A convenient sample was employed by interviewing students with a minimum age of 18, and only those who were willing to provide a written consent to participate in focus group discussions and be tape recorded.

Data were collected using an interview guide. The male student-nurse midwives participated in focus group discussions (FGDs) [20]. The discussions were tape recorded and field notes were written down. Twelve FGDs were conducted. Each focus group constituted 6 to 9 participants. The twelve focus group discussions, managed to give saturated data on the experiences of the male student nurse midwives [21]. One FGD was conducted with year 1 and 2 students while another was conducted with year 3 and 4 students at each nursing college. This helped to gather information on experiences during early and later years of the socialisation process in the nursing education.

### Rigour and trustworthiness

Throughout the study, methods to enhance rigour and trustworthiness were employed through creditability and confirmability [22,23,24,25]. In this study, during all the phases, the researcher followed the appropriate methodologies. A pre-test of the interview guide was conducted at one nursing college with participants who were not included in the study. The pre-test assessed the clarity of the questions, sequence and completion duration. The collected data did not form part of the main study.

Credibility was achieved through tape recording the interviews and keeping notes. This ensured that all useful data were recorded. A male research assistant was utilised during FGDs. The assistant ensured the interviews were being tape recorded and probed where necessary. This helped provide a conducive environment for the male student nurse midwives to recount their experiences.

### Data analysis

The data analysis process utilised the six steps method of thematic analysis [26] with the researcher being the principal data analysis instrument. The six steps are: Familiarising oneself with the data, generating initial codes, searching for themes, reviewing themes, defining and naming themes and finally, producing the report.

Data handling and management was aided with the use of MAXQDA version 11 software.

The tape-recorded data were transcribed verbatim. This allowed the researcher to familiarise oneself with the data. Codes were generated, and categories identified. Common categories were grouped together to formulate and name themes and sub-themes. The themes were reviewed by re-checking the codes, categories and themes. Verification of the data analysis process included editing by re-reading the transcripts while listening to the recorded information, academic supervisors close monitoring of the qualitative data analysis process and later verifying themes that were formulated and member checking. Member checking enhanced confirmability and transparency [27,28]. Further, this helped prevent researcher bias, motivation and perspective [28,29].

## Results

The study revealed that male student nurse midwives had some positive but more negative experiences during their socialization process in this female-dominated profession.

Following the thematic analysis, three major themes were uncovered from challenges experienced by the male student nurse midwives. The following Table 1 gives a thematic map of the findings illustrating the three main themes, sub-themes, and examples of significant statements.

**Table 1:** Thematic Map.

Main Theme	Sub-themes	Examples of Formulated meanings

**Nursing is a feminine occupation**	Men are different from women	- Learning to provide basic patient care was tough, the activities were seen to be feminine
	Feeling Inferior	- Nursing tends to be underrated by many people as such, one felt inferior
	Basic care provision	- Care provision depends on individuals
	Changing or not changing Career	- One’s motivation for the nursing profession decreases with time
**Facing Discrimination**	Sense of belonging or not belonging and sense of not being accepted	- Being underrated by female nurses & students - Female nurse managers favoured female nurses
	A sense of Isolation	- Feeling lonely amidst many female students. - Lack of male role models
**Socialisation process**	Ideal socialisation	- Career guidance helps an individual make an informed choice - Most of the male nurses made an uninformed choice when joining nursing
	Failed socialisation	- Some male nurse midwives qualified without being able to exhibit the expected behaviour of professional nurse midwives.

A ‘thick’ description of each theme and sub theme is provided and discussed.

### Nursing is a feminine occupation

The study findings revealed that most nursing activities are considered feminine in nature. The activities mainly constitute basic care for patients and include: helping patients meet activities of daily living such as feeding; providing bed baths and changing soiled linen among others. Under the theme ‘Nursing is a feminine occupation’, there are four sub-themes explained and discussed below.

The first sub-theme was ‘men are different from women’. The male student nurse midwives perceived themselves to be different from females and that, in the Malawian culture, certain duties were considered exclusively feminine.

The majority of the participants in eight of the 12 FGDs explained that most of the nursing activities in Malawian society were performed by women. It was clear that men joined nursing ignorant of what they were to undertake in the profession. In one FGD the student participants indicated that though nursing was a caring activity, anybody irrespective of an individuals’ gender could provide care on condition that they learnt the required skills. However, in the same FGD, some participants were quick to mention that those who had a passion for the profession did not find it hard performing activities which were considered feminine in nature.

Some participants said:

“…*But to me I would say there are challenges. There are some procedures which you feel you being a man maybe you are not supposed to do that, things like bed bathing. Culturally its women who always do that, even at home…” male student nurse, NMT, FGD 2, year 1, Res 2, Line 95*.

#### A feeling of being inferior

The study revealed that participants in nine of the FGDs, expressed that male student nurse midwives felt inferior to other men working in the health sector. The male student nurse midwives compared themselves with those who could be studying toward registration or working in male dominated health professions such as medical assistants or medical doctors. Similarly, in colleges or universities which offer a variety of courses other than nursing, some male student nurse midwives were teased for having joined a feminine profession by either male students undertaking non-nursing courses or female nursing students or qualified female nurse midwives.

Some participants stated:

“*Like for me when I came here… I had the passion for the sick. But slowly, I found that I was demotivated due to others saying… ‘aah! So, you are a male student nurse midwife. My friend all these programs here at X university you decided to choose nursing why?’” male student nurse, RN, year 2, FGD7, Res5, Line 14*.

Due to feelings of inferiority, a fifth percent of the study participants expressed that they experienced role conflict secondary to cultural expectations. This resulted in some of the male student nurse midwives displaying unprofessional behaviours following loss of motivation in the profession. For example, some senior students would be tempted to jump the ‘nurse’s scope of practice’.

#### Basic care provision

The third sub-theme was ‘basic care provision’. When study participants were asked if there were any differences in terms of care of patients based on care rendered by either a female or male nurse midwife, there were varying responses. Though both male and female nurse midwives cared for patients, there were both positive and negative attitudes towards the care rendered by male nurse midwives. Some study participants stated that some female patients preferred male nurse midwives especially during labour and delivery. This was because some female nurse midwives were perceived to be provocative and that some shouted at the pregnant mothers during labour and delivery unlike male nurse midwives. However, study participants in the three FGDs explained that some female patients refused to be cared for by either student or qualified male nurse midwives. The study participants in one focus group discussion indicated that some community members (especially husbands of some patients) did not like their wives to be attended to by male nurse midwives.

Some of the participants shared this:

“*…in the community, the husbands said their wives shouldn’t be delivered by a male nurse midwife. While the wives said the male nurse-midwives were perfect compared to the female nurse midwives” Male Student nurse, Yr3, FGD, Line 145 Res4*.

#### Changing or not changing career

The forth sub-theme was that of changing or not changing their career. The study participants were asked what they would do if they were given a chance to pursue another career. Responses were varied depending on the course being undertaken whether RN or NMT course. Furthermore, the level/year at which an individual was in the course of training contributed to whether that individual would change or not change career. A majority of the student-nurses undertaking the NMT course in 5 of the 12 FGDs were likely to change career unlike RNs. A majority of male student nurse midwives in three FGDs who were ready to change the career were those in year 3 for the NMTs since this was a senior class unlike the junior classes. Those in senior classes indicated that their motivation for nursing had declined due to what they had experienced in the profession with nurse midwives and medical clinicians. Submissiveness of nurses to medical clinicians, routine work in the clinical area, favouritism toward female nurses by nurse managers, and lack of recognition by some female nurses and lecturers were examples of the reasons indicated that would make an individual abandon the nursing career. A long career path was one of the reasons that would make the NMTs move out of the profession. A majority of the student NMTs shared these sentiments;

Some male nurse midwives had this to say:

“*…Motivation is not all that good now… In first year you find us very motivated, but in 4th year, by and by people start losing that motivation so… in most cases it’s the negative interaction with nurse leaders and lack of recognition. I don’t see myself in this profession for the next 2 years coming. If I will be given chance to change the career I will grab it with both hands.” Male student nurse, FGd7, RN, year 4, Res4, lines 41–42*.

Another said:

“*…We have problems with upgrading. If upgrading from technician to professional level was easy more people would have joined and remained in nursing” Male Student Nurse, FGD5, year 1, NMT, Res, lines 31–32*.

On the other hand, some study participants perceived they would not easily change the career or leave for greener pastures because they were passionate about caring for patients and that there were other benefits that the profession offered. For example, nursing was seen to be very dynamic in that an individual could specialize in any area of interest, limited career choices in other careers and that there was job security. This was evident in that study participants who were older and had worked previously were not ready to change the career having experienced disappointments in other occupations.

### Facing Discrimination

Some study participants experienced a sense of not being accepted and were isolated. Participants in five of the 12 FGDs shared sentiments of being discriminated by female student nurse midwives, lecturers, clinical nurses and sometimes patients. Sub themes that emerged under this theme were ‘a sense of not being accepted’, ‘a sense of belonging or not belonging’ and ‘being isolated’:

#### Sense of belonging or not belonging and ‘a sense of not being accepted’

More than half of the study participants had a sense of not belonging because of perceived discrimination from some of the patients, lecturers and qualified female student nurse midwives.

Some had this to say:

“*…but then you would hear comments to say ‘you are not man enough; why are you studying nursing? Your fellow men are not studying nursing’, so you would think to say what message is she trying to say”, Male student nurse, FGD8, NMT, year 2, Lines 151–2 Res5*.

“*It’s a matter of personality, they would threaten us boys as if maybe we have ventured into their (female nurses) profession. You may hear them say ‘you men you are spoiling our profession’ mukutiwonongera polofeshoni yathu’ (All laughs)…” male student nurse, FGD4, year 1, RN, res 1, Line 20*.

#### A sense of isolation

The third sub theme on facing discrimination was ‘a sense of isolation’. When the study participants were asked on how they viewed implementation of the gender policy in their institutions, some participants explained that the gender policy was good because it facilitated incorporation of men into the nursing profession. However, more than fifty percent of the participants perceived that the policy was not being fully implemented because there was an imbalance at the rate of recruiting females compared to males as more females were recruited. Consequently, this resulted in feelings of being lonely and isolated thereby affecting the male student nurse midwives socially and academically.

This transpired in one of the FGDs as follows:

“…*The other issue is that I can give an example the time we were doing clinical practice I found myself being the only man amongst 10 girls (all laughs)…”. male student nurse, FGD2, Nurse Technician, Year 2, Res 2, Line 28*.

Lack of male role models also contributed to feelings of isolation in male student nurse midwives. The fact that there were very few or no male nurse role models, to whom the male student nurse midwives could look up, was a barrier in the socialization process. Some of the study participants, especially junior students in 3 of the 8 FGDs, explained that it became a problem to associate with nurse midwives of the opposite sex. Consequently, it compromised their learning.

### Socialisation Process

The third theme was socialisation process. The male student nurse midwives transited through three stages of socialisation process. The stages are: Anticipatory socialisation, Formal and Post training [30]. It should be noted that the study participants did not stipulate names of the stages per se but the linchpin of the discussion and coding revealed there were three stages.

There were ideal and failed socialisation processes as sub-themes.

#### Ideal socialisation

Ideal socialisation was presumed to take place when a student joined nursing having made an informed career choice and upon undergoing training, acquired and exhibited the expected characteristics of a nurse midwife. During the process of being socialized into nursing, the student was expected to acquire attributes such as competency, compassion, critical thinking skills, empathy, good communication skills, and patience.

For an individual to acquire the expected professional characteristics there are some contributing factors: Those who made an informed choice to join nursing and had a passion for their chosen career managed to positively handle challenges faced during training [31,32].

#### Failed Socialisation

When the male student nurse midwives joined the profession without making an informed decision, it resulted in loss of interest in the profession and affected their academic performance. This could result in being withdrawn from the course or some would leave and pursue another career. In some cases, some male nurses would behave unprofessionally, such as being in civilian clothes while on duty, in an effort to cope especially after qualifying.

## Discussion

The perception that nursing is a feminine profession is similar to findings in other studies [10,33]. Participants in a qualitative study felt that nursing was a feminine profession [11]. The male students had joined nursing involuntarily due to limited space for entry into other university courses.

In the current study, participants felt that nursing was a feminine profession too. Nonetheless this, in one FGD some participants explained that though nursing is seen as a caring profession, anybody could provide care irrespective of gender. With this in mind, some of the study participants joined the profession, however, more than half of the study participants claimed that they made an uninformed career choice. Not only do they join nursing because they think they can render the care, but they join due to other reasons such as limited career choices, job security and the fact that in nursing there are professional opportunities because it offers varied specialties and flexibility. This has been documented in the literature [7,8,9,10].

Even though there were some positive experiences or benefits in the profession, the male student study participants in the current study reported many challenges. These included feeling inferior, discrimination, isolation and a sense of not belonging. This tends to have a negative effect on how the male student nurse midwives render nursing care thereby compromising care of patients. For example, some senior students were likely to exceed their scope of practice to compensate for their feelings of inadequacy. Coupled with challenges of feeling inferior and cultural expectations for a man, in this study it resulted in loss of motivation and ‘denial of professional identity’. By identifying with the medical profession through role conflict, not wearing the required uniform when on duty as they observed this in the qualified male nurse midwives, and breaking the nurses’ scope of practice, professional ethics could also be compromised.

According to the social role theory, male student nurses are expected to undergo a role change if they are to be fully socialized [19,34]. Though the theory implies that gender differences are not rigid as they depend on one’s immediate social role irrespective of an individual’s sex, over half of the study participants in this study showed that role adaptation in the female dominated profession was difficult. This is evident in some acting outside their scope of practice and identifying with traditionally male dominated professions such as medicine. Considering that Malawi is a patriarchal society, the participants perceived that they had a low status job associated with women [35]. Consequently, the male nurse may fail to recognise and utilize opportunities that may be available in the profession such as job satisfaction and career progression. Interestingly, these opportunities are appreciated by men who join nursing from a previous career, that is having worked elsewhere prior to joining nursing [8,9,36,37]. From these findings it is evident that male nurses are facing many challenges in the nursing profession. For male student nurse midwives, isolation and lack of role models are critical factors to be addressed if they are to be retained in a nursing learning environment or work place upon qualifying. Isolation and lack of male role models for male student nurses during college life has been reported in literature [38,39], which resulted in absenteeism and consequently some failing examinations and others quitting the profession altogether [40].

The positive impact of the information sourced prior to joining the female dominated profession indicated that individuals tended to be psychologically prepared to be trained and retained [8]. In addition, a literature review found that early professional socialisation activities such as career choice and guidance strongly influenced the person’s view of the profession and career decision making. The informed decision helps prepare students psychologically and this enables them to acquire the expected professional attributes.

## Limitations

Though all students may have used the same clinical area for practicals, teaching/learning environments in the respective nursing colleges tended to differ because the RNs were from universities as opposed to students from colleges that educate nurse technicians. This may have exposed the students to different socialisation environments.

## Conclusion

Malawian male student nurse midwives faced positive and more negative experiences during their education in nursing. For example, feeling of being inferior, lack of recognition by some female-nurse midwives, lecturers and patients and discrimination. The majority of student nurses in the study lacked information regarding nursing prior to joining the profession. This also contributed to the challenges encountered. Thus, career guidance should be intensified by utilising various means of gender-inclusive communication strategies. Use of male nurse role models would be ideal in promoting nursing and midwifery profession. This could help prospective male student-nurse midwives, in particular, make an informed career choice.

Nurse educators and clinical nurse midwives should ensure formulation and implementation of gender-inclusive policies in nursing colleges and hospital facilities. This could strengthen the education and retention of men in the female dominated profession.
